# Echo Frequency Estimation Technology for Passive Surface Acoustic Wave Resonant Sensors Based on a Genetic Algorithm

**DOI:** 10.3390/s23239401

**Published:** 2023-11-25

**Authors:** Yufen Wu, Yanling Li, Xue Wang, Jianchao Zhang, Jin Yang

**Affiliations:** 1 College of Physics and Electronic Engineering, Chongqing Normal University, Chongqing 401331, China; 2022210511032@stu.cqnu.edu.cn (Y.L.); 20180802027t@cqu.edu.cn (X.W.); 2 College of Optoelectronic Engineering, Chongqing University, Chongqing 400044, China; 20161802039@cqu.edu.cn

**Keywords:** resonant sensors, surface acoustic wave (SAW), echo frequency estimation, genetic algorithm

## Abstract

Passive wireless surface acoustic wave (SAW) resonant sensors are widely used in measuring pressure, temperature, and torque, typically detecting sensing parameters by measuring the echo signal frequency of SAW resonators. Therefore, the accuracy of echo signal frequency estimation directly affects the performance index of the sensor. Due to the exponential attenuation trend of the echo signal, the duration is generally approximately 10 μs, with conventional frequency domain analysis methods limited by the sampling frequency and data points. Thus, the resolution of frequency estimation is limited. Here, signal time-domain fitting combined with a genetic algorithm is used to estimate SAW echo signal frequency. To address the problem of slow estimation speed and poor timeliness caused by a conventional genetic algorithm, which needs to simultaneously estimate multiple parameters, such as signal amplitude, phase, frequency, and envelope, the Hilbert transform is proposed to remove the signal envelope and estimate its amplitude, and the fast Fourier transform subsection method is used to analyze the initial phase of the signal. The genetic algorithm is thereby optimized to realize the frequency estimation of SAW echo signals under a single parameter. The developed digital signal processing frequency detection system was monitored in real time to estimate the frequency of an SAW echo signal lasting 10 μs and found to have only 100 sampling points. The proposed method has a frequency estimation error within 3 kHz and a frequency estimation time of less than 1 s, which is eight times faster than the conventional genetic algorithm.

## 1. Introduction

Passive wireless surface acoustic wave (SAW) resonant sensors are used in various fields, such as train wheels, artillery, tank tracks, high-voltage electricity, and strong electromagnetic fields, due to their non-contact feature, fast speed, anti-interference aspect, easy coding, small size, and robustness. They do not require a power supply but rely on receiving electromagnetic waves for energy supply to measure physical quantities such as temperature, strain, torque, gas, and pressure [1,2,3,4,5,6,7,8]. According to the detection principle, SAW sensors can be divided into delay types and resonance types. Among the latter, the resonance frequency change in SAW resonators reflects the measured change, and the echo signal contains the measured information of the sensor. Therefore, by analyzing the frequency of the echo signal, changes in the physical characteristics of the sensor can be detected [9,10,11].

However, the characteristics of high frequency, narrow band, exponential decay, and short duration create inconveniences in the measurement of echo signal frequencies. Conventional frequency domain algorithms and hardware counting methods are limited by the signal sampling length and produce significant errors. In addition, modern spectral estimation methods, such as the Music algorithm, Autoregressive (AR) model algorithm, and maximum likelihood estimation algorithm, are limited in further application due to their complexity, large calculation quantity, and difficulty in achieving real-time processing.

This article is focused on the characteristics of SAW sensing signals and uses the time-domain signal fitting method to estimate the frequency parameters of SAW sensing echo signals. The traditional fitting method uses the least squares method for parameter fitting, but its application is limited to linearization problems, leading to a large calculation quantity and low accuracy. Therefore, the genetic algorithm for signal parameter fitting is adopted. However, in the application of classical genetic algorithms [12,13,14,15], it is necessary to clarify multiple parameters of the estimated signal, such as amplitude, phase, frequency, and the envelope attenuation coefficient, which will lead to low fitting efficiency. Herein, the signal was preprocessed to optimize the local operation of the genetic algorithm by analyzing the sensing characteristics of SAW echo signals. Finally, only the optimized genetic algorithm was used to estimate the frequency of the SAW echo signal with a single parameter, thereby improving the fitting speed and meeting accuracy requirements for frequency estimation.

## 2. The Characteristics of a SAW Resonator

**Figure 1:**
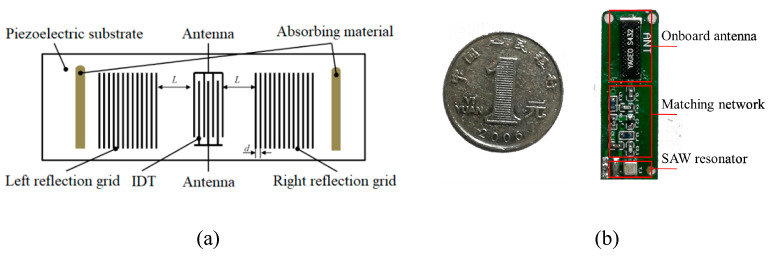
(**a**) Structure of SAW resonant sensor; (**b**) Physical diagram of SAW resonant sensor.

Figure 1a is a structural schematic of the typical SAW device. The SAW resonator converts the electromagnetic wave into a SAW by emitting an interdigital transducer (IDT), and the SAW is converted into an electrical signal after the SAW has propagated a certain distance through the medium to reach the receiving IDT. During the transmission of the SAW, changes in the material and structure of the IDT sensor cause changes in the transmission medium, resulting in changes in the frequency of the SAW. Therefore, measuring changes in the frequency of the SAW enables the detection of sensing parameters.

Figure 1b is a physical diagram of the SAW device. The SAW device consists of a SAW resonator, matching network, and onboard antenna. The resonant frequency of the SAW resonator is 433 MHz, provided by Zhongke Crystal Electronics Co., Ltd. The matching network, comprising of inductors and capacitors, is employed between the antenna and the SAW for the modulation of the sensor signal to the SAW’s response signal and impedance matching. Changes in the matching network parameters will pull the resonance frequency of the SAW and change the matching conditions, thus leading to a returned response signal consisting of a frequency shift and a change in the amplitude.

**Figure 2:**
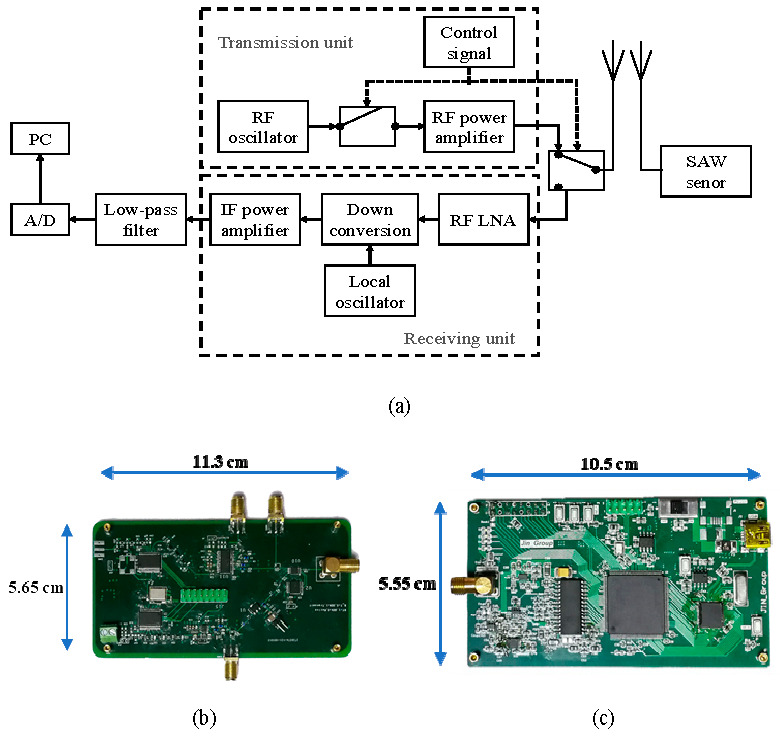
(**a**) Passive wireless SAW sensor measurement system; (**b**) RF interrogator of SAW sensor; (**c**) Data collector of SAW sensor.

Figure 2a shows the SAW resonator system composed of the excitation and sensing signal reception and processing. The radio frequency (RF) oscillator generates a signal of a specified frequency, which is amplified by the RF power amplifier, and sends out an excitation signal through the antenna, which is received by the SAW sensor. The system stops the transmission of excitation signals through a single-pole double throw switch and receives the echo signal from the SAW resonator, which is then amplified by the RF power amplifier with RF down-conversion, IF amplification, low-pass filtering, and A/D acquisition, and finally transmitted to a PC terminal for processing. The SAW RF interrogator and SAW echo signal data collector are shown in Figure 2b and Figure 2c, respectively.

The echo signal received by the receiving unit is mixed with the excitation signal and other noise. The down-conversion changes the high-frequency SAW signal into an intermediate frequency, while the shape envelope of the signal does not change. The sensing signal model x(t) is expressed as [16]:

(1) $$x\left(t\right)=s\left(t\right)+c\left(t\right)+n(t)$$

Among them:

(2) $$s\left(t\right)=A(t)sin(w_{0}t+\sigma )$$

$$A\left(t\right)=me^{-kt+l}$$

(3) $$c\left(t\right)=C\sin\left(w_{c}t+\theta \right)=\mathrm{A cos}\left(w_{c}t\right)+\mathrm{B sin}\left(w_{c}t\right)$$

A=C sinθ,B=C cosθ

(4) $$T=\left(m_{R}+m_{L}+2N\right)\cdot \tau_{0}$$

$$\tau_{0}=1/(2f_{d})$$

where $s\left(t\right)$ is the transient and attenuated sensing signal of the resonator; it is a useful signal containing the characterization of sensing information. The transient duration T is usually approximately 10 μs, and its expression is shown in Formula (4): $m_{R}$ is the interdigital logarithm of the output IDT, $m_{L}$ represents the interdigital interaction logarithm in the transducer, N is the number of interdigital electrode cycles, and $f_{d}$ is the resonant frequency of the resonator. A(t) is the envelope of the sensor signal with an exponential attenuation trend, $w_{0}$ is the frequency of the useful signal, k is the attenuation coefficient, l is the unknown of the attenuation envelope, and σ is the random initial phase. Therefore, it can be seen that the sensor signal model $s\left(t\right)$ contains five unknown parameters. $c\left(t\right)$ is the sinusoidal excitation signal that leaks through the system switch during signal reception. For $s\left(t\right)$, $c\left(t\right)$ is a noise interference that needs to be suppressed. $w_{c}$ is the known excitation frequency, and θ is the random initial phase. n(t) is the additive noise with white noise characteristics generated by the channel.

**Figure 3:**
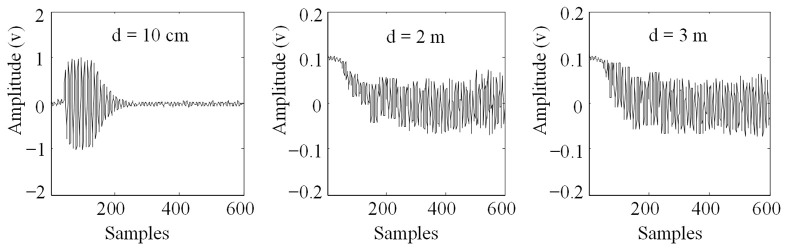
Down-conversion sampling signal of the SAW.

Figure 3 reveals the signal time-domain waveform obtained by different wireless distance d. The sampling frequency of the signal is 10 MHz, and 600 sampling points are intercepted. When d=10 cm, the sensing signal is significantly stronger than the leaking excitation signal and channel noise due to the close distance, so it is the front end of the signal time-domain waveform, and the transient attenuation trend of the sensing signal is clearly visible. However, the useful signal is submerged in the leaking excitation signal and channel noise at the signal of 2~3 m, and there is no obvious attenuation trend.

## 3. The Principle of Single-Parameter Genetic Algorithm Estimation for SAW Resonance Frequency

Since the amplitude, initial phase, frequency, and attenuation envelope information of the SAW echo signal are unknown, when using a genetic algorithm to estimate signal frequency, other parameters except frequency need to be fitted simultaneously, which greatly reduces the fitting efficiency. The research purpose of this article was to estimate the frequency of the signal. Therefore, the amplitude, initial phase, and envelope information of the signal were first estimated, and only a single frequency parameter of the signal was fitted.

### 3.1. Research on a Frequency Detection Method for the Surface Acoustic Wave Echo Signal

**Figure 4:**
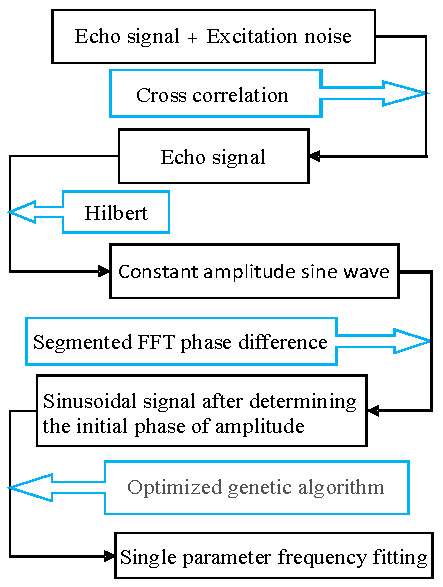
Design process of surface acoustic wave signal processing scheme.

The design process of the surface acoustic wave signal processing scheme is shown in Figure 4. The cross-correlation method was used to suppress excitation noise, and the model was simplified through envelope analysis using the Hilbert transform. Then, the initial phase of the envelope signal was estimated based on the segmented FFT phase difference. Furthermore, the impact of the initial phase error estimated using the single-parameter genetic algorithm on frequency estimation is further investigated. Thus, the optimized genetic algorithm was used to perform single-parameter frequency fitting on the selected signal after determining the initial phase of the amplitude. And the genetic algorithm selection operator was optimized, greatly reducing the complexity and computational complexity of the program, and further improving the genetic selection operator.

### 3.2. Estimation of the Amplitude and Phase of the Sensing Echo Signal

According to Formula (2), a useful signal consists of an envelope signal and a sine signal. Therefore, Hilbert envelope demodulation was used to remove the envelope signal, and the amplitude and initial phase information of the sine signal after envelope removal were analyzed.

#### 3.2.1. Hilbert Complex Analytic Envelope Demodulation

The definition of the Hilbert transform [17] for the real signal $s\left(t\right)$ is as follows:

(5) $$\hat{s}\left(t\right)=H\left\{s\right\}=h\left(t\right)*s\left(t\right)=\int_{-\infty }^{\infty }s\left(t\right)h\left(t-\tau \right)d\tau \;=\frac{1}{\pi }\int_{-\infty }^{\infty }\frac{s(\tau )}{t-\tau }d\tau$$

where h(t)=1⁄πt, and its frequency domain characteristics are:

(6) $$H\left(j\omega \right)=F\left(\frac{1}{\pi }\right)=-j\;sgn\left(\omega \right)\;sgn\left(\omega \right)=\left\{\begin{array}{c} 1,\;\;\omega \geq 0\\ -1,\;\;\omega <0 \end{array}\right.$$

That is:

(7) $$\hat{S}\left(j\omega \right)=H\left(j\omega \right)\cdot S\left(j\omega \right)=-j\;sgn\left(\omega \right)\cdot S\left(j\omega \right)$$

Thus, the Hilbert transform of the real signal is equal to the output response of the signal after passing through a linear system with an impulse response ht=1πt. After the Hilbert transform, the amplitude of each frequency component in the frequency domain remains unchanged, and the phase shifts 90°; that is, the positive frequency lags π/2 and the negative frequency leads π/2, respectively. Therefore, the Hilbert converter is called a 90° phase shifter.

The analytical signal of $s\left(t\right)$ is expressed as:

(8) $$\overset{ˇ}{s}\left(t\right)=s\left(t\right)+\hat{s}\left(t\right)=Ke^{j\varphi (t)}$$

The mode of $\overset{ˇ}{s}(t)$ is the envelope of $s\left(t\right)$:

$$K=\left|\overset{ˇ}{s}\left(t\right)\right|$$

After removing the envelope, the real signal becomes a sine wave of equal amplitude:

(9) $$s\left(t\right)=sin(w_{0}t+\sigma )$$

#### 3.2.2. Segmented FFT Initial Phase Analysis

Suppose the expression of the real signal $s\left(t\right)$ is

(10) $$s\left(t\right)=a\cdot cos(2\pi f_{0}t+\sigma )$$

where $f_{0}$ and σ are the amplitude, frequency, and initial phase of the sinusoidal signal, respectively. Set the sampling time as T, the sampling number as N, and sampling interval $T_{0}=T/N$; then, the sampling sequence of real signal $s\left(t\right)$ is

(11) $$s\left(nT_{0}\right)=a\cdot cos(2\pi f_{0}nT_{0}+\sigma ),n=0,1,2,\ldots ,N-1$$

The discrete Fourier transform is performed on $s\left(nT_{0}\right)$:

(12) $$S_{k}=\frac{a\cdot \sin[\pi \left(k-f_{0}T\right)]}{2\cdot \sin[\frac{\pi }{N}\left(k-f_{0}T\right)]}\cdot e^{j[\sigma -\frac{N-1}{N}\pi \left(k-f_{0}T\right)]},k=0,1,2,\ldots ,\frac{N}{2}-1$$

Here, only the positive half axis of the frequency is analyzed; that is, the first half of N points of the discrete spectrum are analyzed. The phase information $\sigma_{k}$ of $S_{k}$ is

$$\sigma_{k}=\sigma -\frac{N-1}{N}\pi \left(k-f_{0}T\right)$$

Set $\hat{k}$ as the maximum spectral line, and the phase information $\hat{\sigma_{k}}$ at the same time is

(13) $$\hat{\sigma_{k}}=\sigma -\frac{N-1}{N}\pi \left(\hat{k}-f_{0}T\right)$$

$\hat{k}-f_{0}T=∆k$; ∆k represents the deviation between the maximum spectrum line and the actual spectrum line, and the range is −0.5 to 0.5.

The sampling sequence of the real signal $s\left(t\right)$ is divided into two equal length sequences, $s_{1}\left(t\right)$ and $s_{2}\left(t\right)$. For the convenience of calculation, the number of data points of $s\left(t\right)$ is set as 2N; then, the number of data points of sequence $s_{1}\left(t\right)$ and $s_{2}\left(t\right)$ are both N. The expression of $s_{1}\left(t\right)$ and $s_{2}\left(t\right)$ is as follows:

(14) $$s_{1}\left(t\right)=a\cdot cos\left(2\pi f_{0}t+\sigma \right)=a\cdot cos(2\pi f_{0}t+\sigma_{1})$$

(15) $$s_{2}\left(t\right)=a\cdot cos\left[2\pi f_{0}\left(t+T\right)+\sigma \right]=a\cdot cos(2\pi f_{0}t+\sigma_{2})$$

$$\sigma_{1}=\sigma ,\;{\;\sigma }_{2}=\sigma +2\pi f_{0}T$$

According to Formula (13), after the discrete Fourier transformation of signals $s_{1}\left(t\right)$ and $s_{2}\left(t\right)$, the phases obtained are respectively:

(16) $$\hat{\sigma_{k1}}=\sigma_{1}-\frac{N-1}{N}\pi \left(\hat{k}-f_{0}T\right)=\sigma_{1}-\frac{N-1}{N}\pi ∆k$$

(17) $$\hat{\sigma_{k2}}=\sigma_{2}-\frac{N-1}{N}\pi \left(\hat{k}-f_{0}T\right)=\sigma_{2}-\frac{N-1}{N}\pi ∆k$$

Subtract Formula (17) from Formula (16) to obtain:

(18) $$\hat{\sigma_{k2}}-\hat{\sigma_{k1}}=\sigma_{2}-\sigma_{1}=2\pi f_{0}T=2\pi \hat{k}-2\pi ∆k=-2\pi ∆k$$

From the range of *k*, it can obtain the range of $\hat{\sigma_{k2}}-\hat{\sigma_{k1}}$ is (−π,π).

Substitute Formula (18) into Formula (16) to obtain:

(19) $$\sigma =\sigma_{1}=\hat{\sigma_{k1}}-\frac{N-1}{N}\cdot \frac{\hat{\sigma_{k2}}-\hat{\sigma_{k1}}}{2}$$

σ is the initial phase of $s\left(t\right)$.

### 3.3. Single-Parameter Genetic Algorithm Estimation of the Signal Frequency

When genetic algorithms solve problems, they first encode the solution to form an individual. Different individuals form a population, and the fitness function is determined according to the objective function, which then makes the population evolve into a new generation of a better population through three operators: selection, crossover, and mutation. Evolution continues in this way until a solution that meets the requirements is found. This article presents local optimization on traditional genetic algorithms and uses the upper bound deterministic selection method for replication operations to improve the iteration efficiency of genetic algorithms.

#### 3.3.1. Determine Selection Operator

The selection (replication) operation plays a crucial role in genetic algorithms, as it helps individuals in a population survive and eliminate the fittest, constantly approaching the optimal solution. The quality of the selection operator directly affects the calculation results of genetic algorithms. Classical genetic algorithms typically use roulette wheel selection as the selection operator [12,15], but the error is large, and it easily falls into local optima. Through comparison, it was found that the deterministic selection method had the best effect. The deterministic selection method includes two methods: upper limit determination and lower limit determination. Here, the upper limit deterministic selection method was used to achieve selection operations, which can achieve better results. The specific implementation process is as follows:

(1) The survival expectation number of individual j in a population with size N is $E_{j}=N\frac{F_{j}}{\sum_{k=1}^{N}F_{k}}$, and $\left\lceil E_{j}\right\rceil$ is the fitness function value of the jth individual.
(2) The determined survival number of the jth individual selected into the next generation population is $\left\lceil E_{j}\right\rceil$; then, the number of individuals in the next generation population is $M=\sum_{j=1}^{N}\left\lceil E_{j}\right\rceil$, and $\left\lceil E_{j}\right\rceil$ is $E_{j}$ rounded upward.
(3) M individuals are arranged in descending order according to the value of the fitness function, and the first N individuals are selected.

#### 3.3.2. Optimization of the Fitness Function

The fitness function is a standard used to distinguish between good and bad individuals in a group, which promotes the evolution direction of genetic algorithms and is used to simulate natural species selection. What is more, the function value of the fitness function is always positive, and its value is larger, which indicates the higher superiority. Individuals with higher fitness function values are more likely to be passed on to the next generation.

The Hilbert transform removes the signal envelope and transforms the attenuation curve fitting problem into a sine curve fitting problem. Sinusoidal curve fitting is a measurement method based on time-domain least squares error analysis, which can obtain the accurate value of the sum of squared residuals of sinusoidal parameters.

The sine curve fitting model is represented as

(20) $$s\left(nT_{0}\right)=Asin(2\pi f_{0}nT_{0}+\sigma )$$

where A is the signal amplitude, σ is the initial phase, $T_{0}$ is the sampling time interval, and $f_{0}$ is the signal frequency. According to the criterion of minimum fitting residual error,

(21) $$\rho =\sum_{i=1}^{n}{\left[x_{i}-Asin(2\pi f_{0}nT_{0}+\sigma )\right]}^{2}$$

When the mean square error ρ is the minimum, the estimated parameter is the characteristic parameter of the signal.

The fitness function ε was determined according to fitting residual Formula (22):

(22) ε=m−ρ

where m is a constant and m >ρ; m=600 was selected in this paper.

## 4. Experimental Data Analysis

### 4.1. Hilbert Envelope Demodulation Analysis

**Figure 5:**
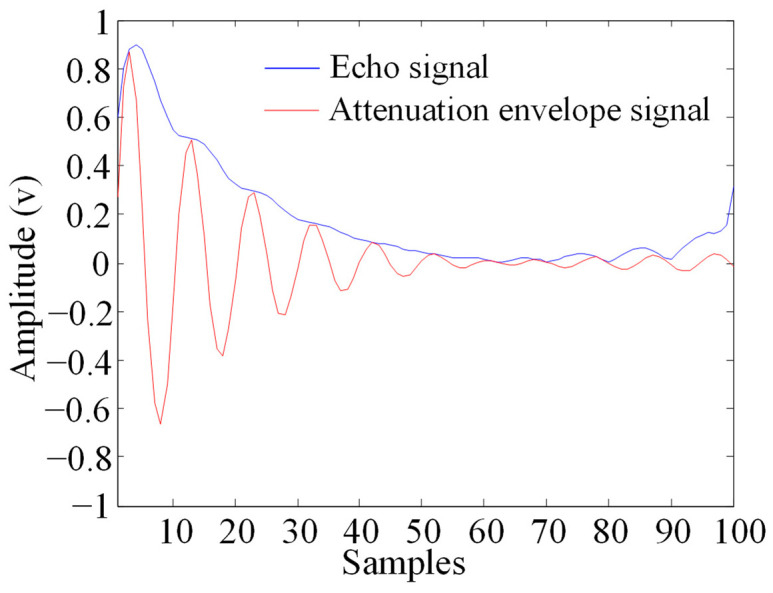
Echo signal and attenuation envelope signal.

**Figure 6:**
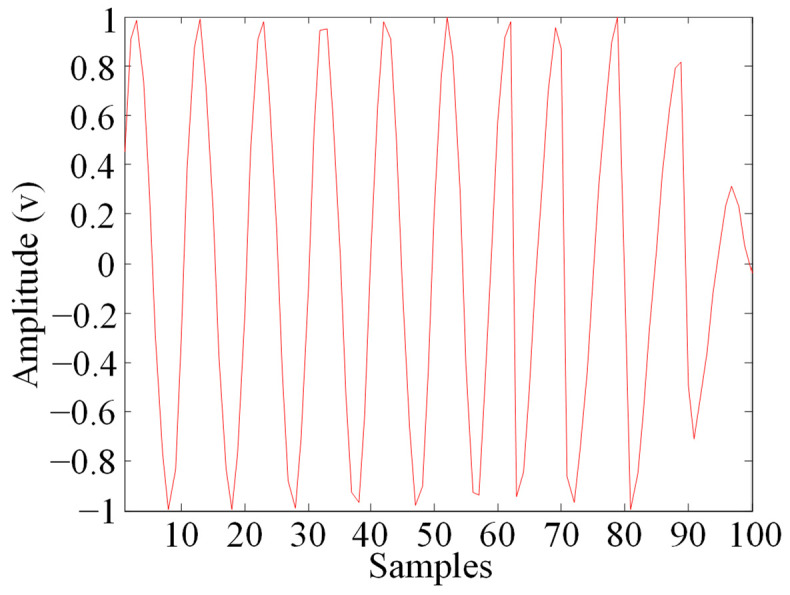
Echo signal after removing attenuation envelope.

The SAW signal with wireless distance d=10 cm in Figure 3 was analyzed, and the attenuated signal with a duration of approximately 10 μs between 100 and 200 sampling points was intercepted. Cross-correlation [18,19] was used to remove mixed excitation noise in the signal. Then, Hilbert complex analytic envelope demodulation was used to de-envelope the useful signal after removing the noise. Figure 5 shows the envelope obtained, where the blue line is the envelope of the echo signal, and Figure 6 is the echo signal after de-enveloping.

After removing the envelope via Hilbert complex analytic envelope demodulation, it was observed that due to the incomplete removal of excitation noise, there was an error in the amplitude of the tail end of the de-enveloped echo signal. To further simplify the signal model, it was assumed that the signal after removing the envelope was a constant amplitude sine wave with an amplitude of 1.

### 4.2. Segmented FFT for Signal Phase Estimation

**Figure 7:**
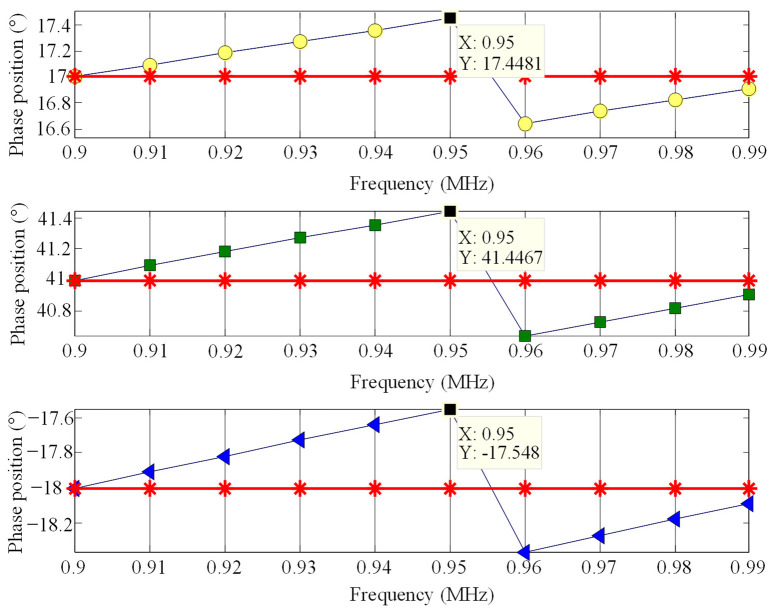
Effect of segmented FFT initial phase estimation.

Sinusoidal signals from 0.90 to 0.99 MHz with an interval of 0.01 MHz were selected. The sampling rate of 10 MHz was used to sample 100 points of selected signals; the signal amplitudes of each frequency were identical, and all were 1. Three initial phases were set for testing the signals of each frequency [20], which were randomly set at 17°, 41°, and −18°. The initial phase estimation results are shown in Figure 7.

In Figure 7, each image sequentially represents the initial phase estimation of signals at different frequencies with initial phases of 17°, 41°, and −18°. The red part represents the true initial phase value, while the remaining color parts represent the phase estimation value using segmented FFT. In Figure 7, it is easy to see that the maximum error of phase estimation at each frequency occurs at 0.95 MHz, and the maximum error of three initial phase estimations at each frequency is approximately 0.45°.

### 4.3. Upper Bound Deterministic Selection Method for Iterative Algebra

Sine signals with an interval of 0.01 MHz from 0.90 MHz to 1.03 MHz were selected, and 100 points of the selected signal were sampled at a 10 MHz sampling rate, as well as with the unchanged amplitude and phase for signals under each frequency.

**Figure 8:**
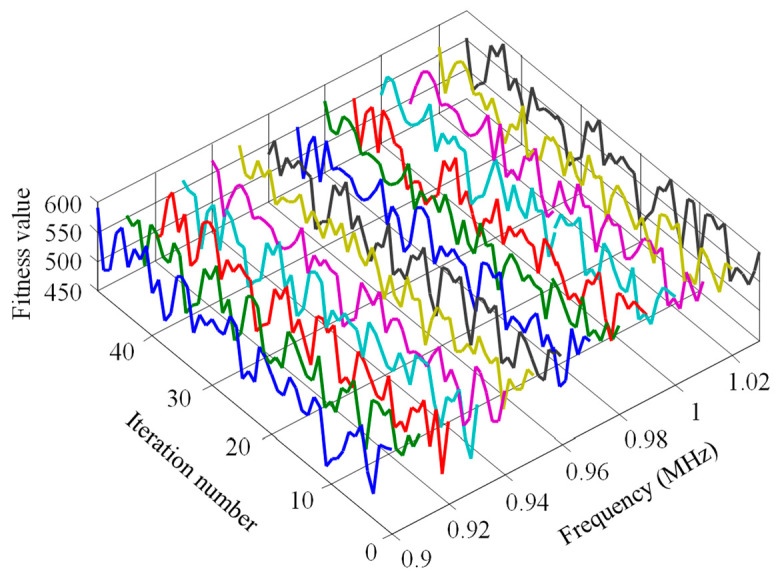
Iterative effect of roulette wheel selection operator.

**Figure 9:**
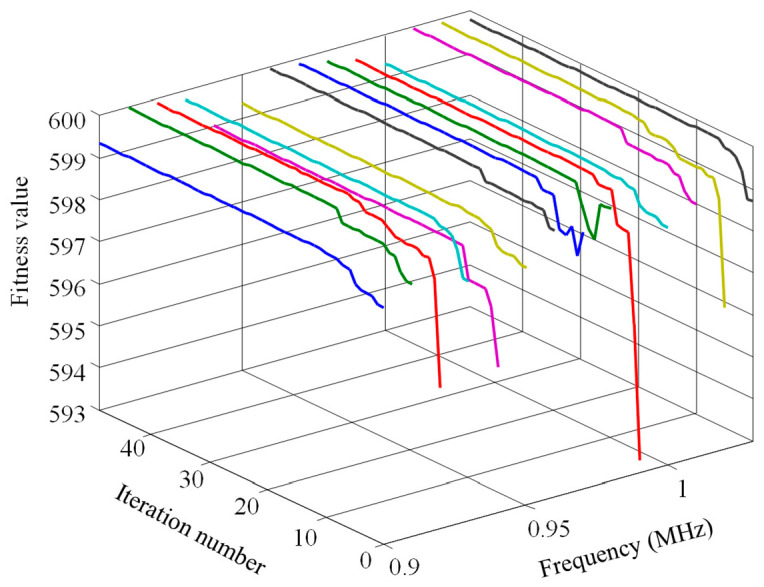
Iterative effect of upper bound deterministic selection operator.

Roulette wheel selection and the upper bound deterministic selection method were used to observe the iterative algebra. Then, the crossover probability was set as 0.6, mutation probability as 0.02, chromosome number as 20, and encoding method as binary. The experimental results are shown in Figure 8 and Figure 9. Among them, the line’s color of Figure 8 and Figure 9 is only to distinguish the curves at each frequency.

By comparing Figure 8 and Figure 9, the fitness function value curve is disorderly and random without a clear trend for when the roulette wheel selection was used. Conversely, the iteration curve shows a regular upward growth trend for when the upper bound deterministic selection method was used for iteration, reaching the maximum value of fitness function in about the sixteenth generation. With the increase of algebra, the value of the fitness function remains basically unchanged.

### 4.4. Accuracy Analysis of Genetic Algorithm Frequency Estimation

**Figure 10:**
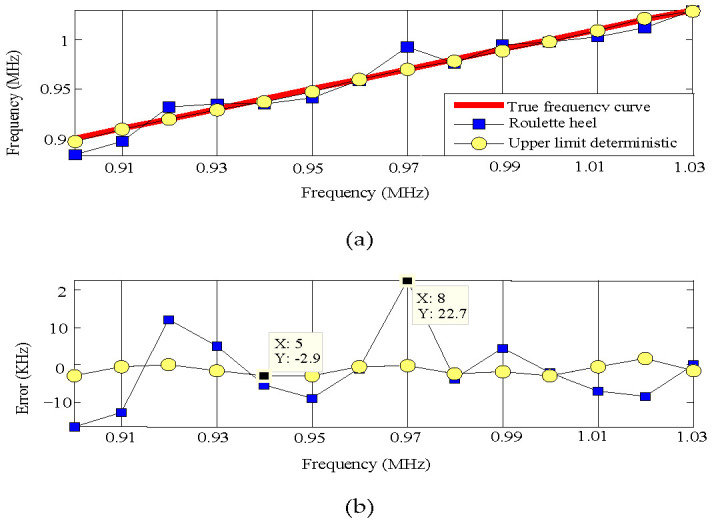
(**a**) Frequency estimation effects of roulette selection and upper bound deterministic selection; (**b**) Frequency estimation errors of roulette selection and upper bound deterministic selection.

Signals ranging from 0.9 to 1.03 MHz with frequency intervals of 0.01 MHz were used for denoising and de-enveloping to determinate the amplitude, as well as segmented FFT analysis to determine their initial phase, and genetic algorithms were then used for frequency estimation. The frequency estimation by the genetic algorithm of the replication operation, using the upper bound deterministic selection method at the sixteenth genetic algebra, and the frequency estimation by the genetic algorithm of the replication operation, using the roulette selection method at the twenty-ninth genetic algebra, are shown in Figure 10. It should be noted that the settings of genetic algebra need to ensure that the maximum fitness function value can be reached by using different selection operators. Therefore, the crossover probability was set as 0.6, the mutation probability as 0.02, the chromosome number as 20, and the encoding method as binary.

Figure 10a shows the frequency estimation effects of two selection operators, while Figure 10b shows the frequency estimation errors of the two selection operators. The roulette wheel selection for copying operations was used, the frequencies ranging from 0.9 to 1.03 MHz had an ~22 KHz maximum frequency estimation error, and the estimation effect was unstable. When using the upper bound deterministic selection method for replication operations in genetic algorithms, the frequencies ranging between 0.9 and 1.03 MHz had a ≤3 KHz frequency estimation error.

### 4.5. Comparison of Single-Parameter and Multi-Parameter Genetic Algorithms

As shown in Table 1, in terms of frequency accuracy, the single-parameter genetic algorithm is approximately seven times more accurate than the multi-parameter genetic algorithm. In terms of genetic algebra, the fitness function value curve of multi-parameter genetic algorithms is disorderly. Because there is high randomness and not a clear trend, the iteration algebra corresponding to the maximum fitness function value may not be clearly determined. However, single-parameter genetic algorithms reach the maximum fitness function value at the sixteenth generation. As the number of iterations increases, the fitness function value remains basically unchanged. Therefore, single-parameter genetic algorithms not only have a regular iteration curve to follow but also very high iteration efficiency. In terms of running time, the running time of the multi-parameter genetic algorithm in DSP is eight times longer than that of the single-parameter genetic algorithm.

It should be noted that a frequency detection system with low power and a 200 MHz main frequency (TMS320VC5509A chip as the core) was built. The running times of the two genetic algorithms were estimated using the software Code Composer Studio V5, which determined the times by calculating the number of CPU clocks.

## 5. Conclusions

In this study, a single-parameter genetic algorithm was used to estimate the frequency of SAW echo signals in response to the challenge of frequency detection in echo signals for passive wireless resonant sensors. By estimating the amplitude and initial phase of removing the envelope signal, it achieved the goal of estimating the frequency of only a single parameter. The selection operator of the genetic algorithm was optimized, which greatly reduced the program complexity as well as the amount of calculation, and improved the efficiency of the algorithm. Hilbert complex analytic envelope demodulation technology was used to remove the envelope part without frequency information, obtaining a constant amplitude sine wave and simplifying the useful echo signal model. Second, the disadvantages of conventional frequency detection technology were analyzed, and a frequency estimation technique based on a genetic algorithm was used. In addition, in order to solve the problem of low efficiency caused by the simultaneous fitting of multiple parameters in the standard genetic algorithm, the amplitude of the useful echo signal after de-enveloping was estimated, and the initial phase was evaluated via the segmented FFT phase difference method to realize the single-parameter frequency estimation. Finally, the standard genetic algorithm was optimized, and the roulette wheel selection was replaced with the upper bound deterministic selection method as the selection operator to further improve the performance of the frequency estimation algorithm. The frequency estimation accuracy of the upper bound deterministic selection method was within 3 KHz, and, compared to the standard multi-parameter fitting genetic algorithm, the fitting efficiency was improved by eight times under the same platform conditions.

## Figures and Tables

**Table 1:** Comparison of single-parameter and multi-parameter genetic algorithms.

Type	Accuracy of Frequency Estimation	Genetic Algebra	Running Time
Single-parameter genetic algorithm	error ≤ 3 KHz	16th generation	0.952 s
Multi-parameter genetic algorithm	error ≤ 22 kHz	irregularity	7.975 s

## Data Availability

The data presented in this study are available on request from the corresponding author.

## References

[1] Kang A., Lin J., Ji X. (2012). A high-sensitivity pressure sensor based on surface transverse wave. Sens. Actuators A Phys..

[2] Martin G., Berthelot P., Masson J. Measuring the Inner Body Temperature using a Wireless Temperature SAW-sensor-based System. Proceedings of the 2007 Ultrasonics Symposium.

[3] Bardong J., Aubert T., Naumenko N. (2013). Experimental and theoretical investigations of some useful langasite cuts for high-temperature SAW applications. IEEE Trans. Ultrason. Ferroelectr. Freq. Control.

[4] Bell D.L.T., Li R.C.M. (1976). Surface-acoustic-wave resonators. Proc. IEEE.

[5] Guo J., Luo Z., Liu B. Discrimination of echo signal of acoustic surface wave resonator. Proceedings of the 2017 Symposium on Piezoelectricity, Acoustic Waves, and Device Applications (SPAWDA).

[6] Wang W., Xue X., Fan S. (2020). Development of a wireless and passive temperature-compensated SAW strain sensor. Sens. Actuators A Phys..

[7] Liu B., Han T., Zhang C. (2015). Error correction method for passive and wireless resonant SAW temperature sensor. IEEE Sens. J..

[8] Rodríguez-Madrid J.G., Iriarte G.F., Williams O.A. (2013). High precision pressure sensors based on SAW devices in the GHz range. Sens. Actuators A Phys..

[9] Pohl A., Ostermayer G., Reindl L. Monitoring the tire pressure at cars using passive SAW sensors. Proceedings of the 1997 IEEE Ultrasonics Symposium Proceedings, an International Symposium (Cat. No. 97CH36118).

[10] Mandal D., Banerjee S. (2022). Surface acoustic wave (SAW) sensors: Physics, materials, and applications. Sensors.

[11] Dixon B., Kalinin V., Beckley J. A second generation in-car tire pressure monitoring system based on wireless passive SAW sensors. Proceedings of the 2006 IEEE International Frequency Control Symposium and Exposition.

[12] Qiu M., Ming Z., Li J. (2015). Phase-change memory optimization for green cloud with genetic algorithm. IEEE Trans. Comput..

[13] Lee Y.H., Park S.K., Chang D.E. (2006). Parameter estimation using the genetic algorithm and its impact on quantitative precipitation forecast. Annales Geophysicae.

[14] Arabali A., Ghofrani M., Etezadi-Amoli M. (2012). Genetic-algorithm-based optimization approach for energy management. IEEE Trans. Power Deliv..

[15] Tuhus-Dubrow D., Krarti M. (2010). Genetic-algorithm based approach to optimize building envelope design for residential buildings. Build. Environ..

[16] Wen Y.M., Li P., Yang J., Zheng M. (2004). Detecting and evaluating the signals of wirelessly interrogational passive SAW resonator sensors. IEEE Sens. J..

[17] Mitra S.K. (2011). Digital Signal Processing: A Computer-Based Approach.

[18] Shahriar M.R., Borghesani P., Randall R.B. (2017). An assessment of envelope-based demodulation in case of proximity of carrier and modulation frequencies. Mech. Syst. Signal Process..

[19] Zhang Y., Xu C., Zhao B. Frequency evaluation of SAW torque response signal using Hilbert envelope-demodulation. Proceedings of the 2010 3rd International Congress on Image and Signal Processing.

[20] Guoqing Q. Digital signal processing in FMCW radar marine tank gauging system. Proceedings of the Third International Conference on Signal Processing (ICSP’96).

